# Assessing the impact of the TB response in Taiwan – the journey towards ending TB

**DOI:** 10.5588/ijtldopen.25.0103

**Published:** 2025-05-12

**Authors:** P.-C. Chan, C.-Y. Chiang, P.-H. Lee, H.-Y. Lo, P.-W. Chu, J.-J. Chen, S. Kato, M.C.B. Raviglione

**Affiliations:** ^1^ Division of Chronic Infectious Diseases, Centers for Disease Control, Taiwan;; ^2^ Institute of Epidemiology and Preventive Medicine, College of Public Health, National Taiwan University, Taipei, Taiwan;; ^3^ Department of Pediatrics, National Taiwan University Hospital, National Taiwan University, College of Medicine, Taipei, Taiwan;; ^4^ Department of Internal Medicine, Wan Fang Hospital, Taipei Medical University, Taipei, Taiwan;; ^5^ Department of Internal Medicine, School of Medicine, College of Medicine, Taipei Medical University, Taipei, Taiwan;; ^6^ International Union against Tuberculosis and Lung Disease, Paris, France;; ^7^ Division of Preparedness and Emerging Infectious Diseases, Centers for Disease Control, Taiwan;; ^8^ Research Institute of Tuberculosis, Japan Anti-Tuberculosis Association, Japan;; ^9^ MACH Centre for Multidisciplinary Research in Health Science. University of Milan, Italy.

**Keywords:** tuberculosis, End TB strategies, policy implementation, TB elimination

## Abstract

The incidence of TB in Taiwan declined by 62% from 2005 to 2023 (i.e., from 73/100,000 to 28/100,000). Here we review the past two decades of TB epidemiology, policy implementation, and outcomes, identifying gaps and solutions for domestic and global responses. An external review in 2024 assessed National TB Program progress towards the End TB goal, integrating feedback from an International Review Panel and a 2023 expert questionnaire. The findings informed Phase III (2026–2030) of the ‘End TB by 2035 Project’. We present review materials, consensus recommendations, and follow-ups through 2024. In 2023, 64% of the TB cases were aged ≥ 65. TB incidence among those < 60 is projected to meet the End TB targets (<10/100,000) by 2035, while elimination (<1 per million) is expected among 0–14-year-olds. During 2005-2024, Taiwan universally adopted new diagnostic tools for drug-resistant TB, shorter regimens and user-friendly platforms for reporting and case management. Nationwide policy innovations included active case finding, and TB infection (TBI) treatment. Taiwan’s consistent investment in TB reflects strong political commitment to End TB. Current challenges include aging, co-morbidities, high TB/TBI among foreign migrant workers and societal disparities, and we suggest that future efforts must leverage artificial intelligence, universal genotyping and greater inter-departmental collaboration.

## INTRODUCTION

Elderly individuals consistently exhibit higher TB incidence rates than younger adults, primarily due to reactivations.^1,2^ TB mortality remains disproportionately high in this age group; in the U.S., 7% of elderly patients died at diagnosis and 21% died during treatment, compared to 2% and 7%, respectively, among younger adults.^2^ This poor prognosis results from non-specific symptoms, reduced drug tolerance, coexisting comorbidities, all contributing to TB transmission in medical institutes and long-term care facilities (LTCF), higher all-cause mortality and lower treatment success.^1,3^ Additionally, increased population mobility from endemic countries represents challenges for TB elimination in low-incidence countries.^4^ To optimize TB care and prevention in migrants, improved coordination is essential, ensuring a balance between individual rights and public health requirements. Strengthening migrant TB control strategies can contribute to Global End TB strategy by 2035.^5^

Taiwan’s unique demographic and public health infrastructure has strongly shaped its TB control efforts. Since the Japanese occupation in 1906, Taiwan has maintained a household registration system, complemented by a national identification system established in 1947. Through such system information is constantly available. By the end of 2023, Taiwan’s population reached 23.4 million, with only 135,571 newborns, making it the lowest in its history.^6^ Fertility rates have dropped from 7 births per woman in 1951 to 1.09 in 2023, making Taiwan one of the world’s lowest fertility-rate countries.^7^ The aging population, accounting for 18% in 2023, is projected to reach 20% by 2025, classifying Taiwan as a super-aged society.^8^

Taiwan achieved a 62% reduction in TB incidence, dropping from 73 cases per 100,000 in 2005 to 28 in 2023, with an average annual decline of 6% (2016–2023).^9^ TB mortality also declined, from 4.3 per 100,000 in 2005 to 1.9 in 2023. COVID-19 pandemic accelerated this decline, with annual reductions of 10.8% in 2020 compared to 2019, and 12.1% in 2021 compared to 2020. By 2022, the decline returned to the pre-COVID-19 trend (5.7%).

The aim of this report is to describe the epidemiological, response and policy context for TB control in Taiwan over the past two decades, to identify barriers to achieving further TB reduction within the next 10 years and outlines strategies to overcome these issues.

## TB CONTROL IN TAIWAN

Since 2006, Taiwan’s National TB Program (NTP) has implemented milestone initiatives, starting with the first National Strategic Plan (NSP) “Ten-Year Halving Tuberculosis Plan” (2006-2015), followed by Phase I (2016-2020) and Phase II (2021-2025) of the “ Taiwan’s End TB by 2035 Project”, reflecting Taiwan's commitment to end TB.^10,11^ Preparations for Phase III (2026–2030) are currently underway. In 2013, the Taiwan Centers for Disease Control (Taiwan CDC) conducted an external review of the NTP to assess the effectiveness of the 2006–2015 Plan.^12^ Based on recommendations from the International Tuberculosis Review Panel (ITRP), Taiwan CDC developed Phase I (2016–2020) of the End TB project. A second review was planned for 2024.

To support the 2024 review, Taiwan CDC conducted an online questionnaire in late 2023, inviting input from TB experts across all levels. Between January and March 2024, detailed analyses of the collected data were prepared. A consensus meeting held on February 27, 2024, by the Ministry of Health and Welfare (MoHW), facilitated discussions on critical challenges and insights for Phase III development. The second external review was conducted in April 2024 to provide guidance for revising the program, addressing evolving epidemiological trends, demographic transitions, emerging technologies, and system and financial challenges.^13^ The review consisted in the following activities: presentation by Taiwan CDC staff, followed by focused discussions; examination of data and re-evaluation; visit to a hospital for DR-TB care, a public health bureau, a district health center and meeting with staff; preparation of a consensus report and presentation to the MoHW.

This collaborative approach ensured that input from both local experts and national policymakers shaped the NSP’s revisions. At the end of the review, the ITRP finalized strategic and technical recommendations to align the NSP with Taiwan’s commitment to achieving the End TB 2035 target.

### A brief history of TB control

The Taiwan CDC, established in 1999, oversees TB control. In 2001, the Chronic Disease Control Bureau (CDCB), previously responsible for TB control in Taiwan, was merged into Taiwan CDC. Taiwan’s NTP is funded entirely by the central government, which reflects the strong political commitment. Since 2003, TB diagnosis has required three sputum samples for smear microscopy and culture for *Mycobacterium tuberculosis complex* (MTBC), ensuring bacteriological confirmation. Under the Communicable Disease Control Act, physicians are mandated to report TB cases, with non-compliance penalties ranging from USD 3,000–15,000.^14^ If a physician employed by a medical institution fails to report in a timely manner, the institution itself is also subject to fines ranging from USD 10,000–66,666.^14^ To support timely notification, Taiwan CDC employs three integrated systems: Notifiable Infectious Disease Reporting System (NIDRS) for case reporting; Laboratory Automated Reporting System (LARS) for linking laboratory data; National TB Management System: a web-based registry connecting public health officials and healthcare providers to manage TB and TB infection (TBI) cases, is smart-card required, password protected and internet protocol (IP) address-sensitive. Taiwan’s National Health Insurance (NHI) provides universal health coverage, ensuring care for 99% residents. To encourage comprehensive reporting, the ‘No Reporting, No Reimbursement’ policy, introduced in 1997, prevents reimbursement for anti-TB care without notification.^15^

Consequently, TB detection peaked in 2004 with 16,784 cases (74.1/100,000).^16^ The NHI reimburses medical services for active TB or TBI patients, ensuring timely access to care without financial hardship. Copayment fees for both TB and TBI patients are reimbursed by Taiwan CDC. For uninsured patients, Taiwan CDC covers all health care related expenditures of TB. Studies revealed relatively short patient delay and health system delay.^17^ TB patients can access services at all hospitals and 89% of private clinics contracted under NHI.^18^

Diagnosis of TB typically begins with a Chest x-ray (CXR) followed by sputum examinations. Approximately 700 TB case managers under NHI through a Pay-for-Performance (P4P) program for TB and bridge clinical care and public health systems, coordinating care and managing challenging cases.^19,20^ Once TB patients are notified, 2,500 public health nurses (PHNs) at 374 public health centers provide health education to TB patients, monitor treatment adherence, conduct contact and outbreak investigations. Regular training programs for clinicians ensure high-quality care by professional TB societies. Former TB supervisors of the CDCB continue to support monthly TB meetings at county and city level. A critical mass of clinicians and researchers committed to the fight against TB was in place, who collaborated with public health sectors in ensuring rational use of anti-TB medicines, especially fluoroquinolones,^21^ which are further ensured through regularly updates of TB guidelines and collaboration between Taiwan CDC and the NHI Administration.^22^

### Epidemiological characteristics of TB in Taiwan

**Figure 1.**
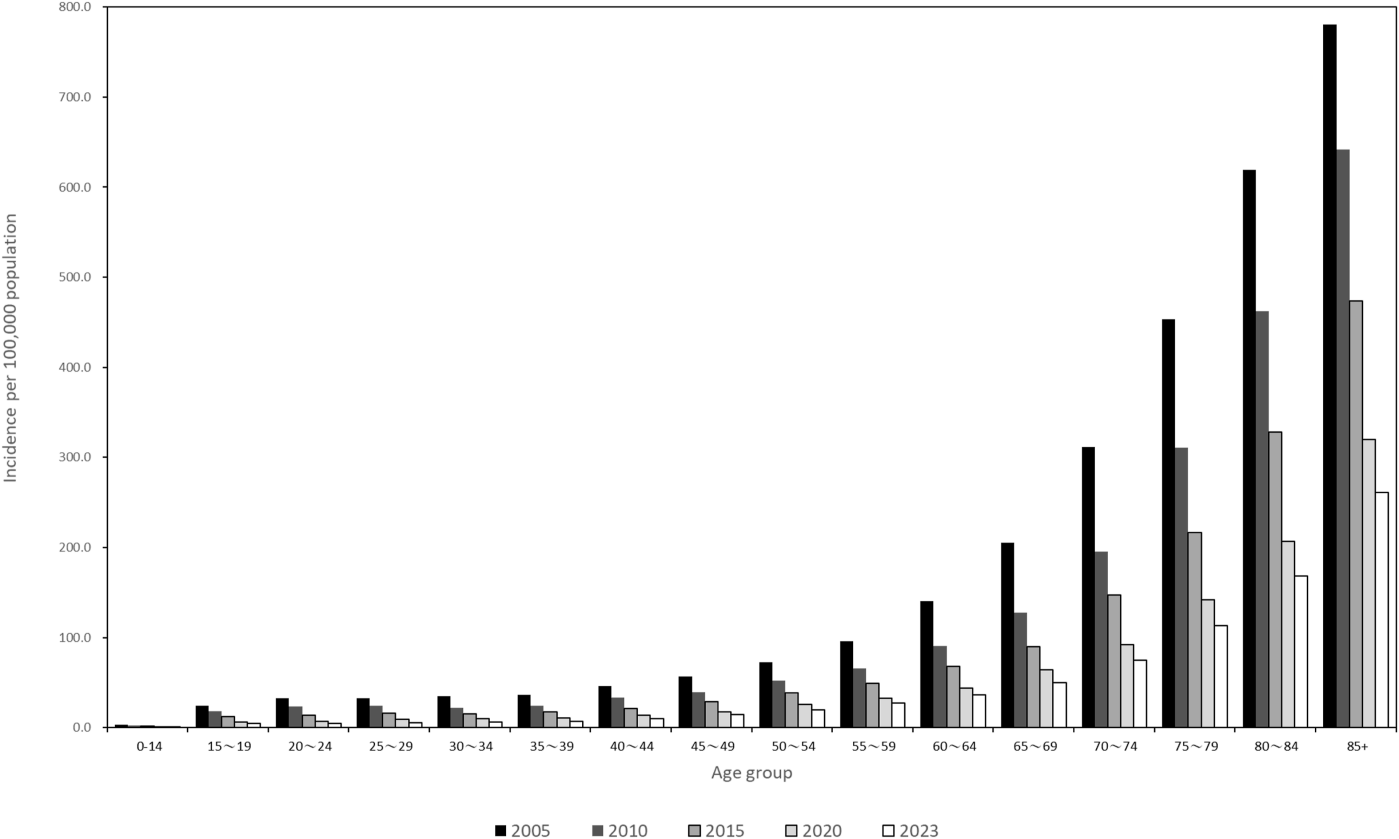
TB incidence rates stratified by age groups, 2005–2023.

**Figure 2.**
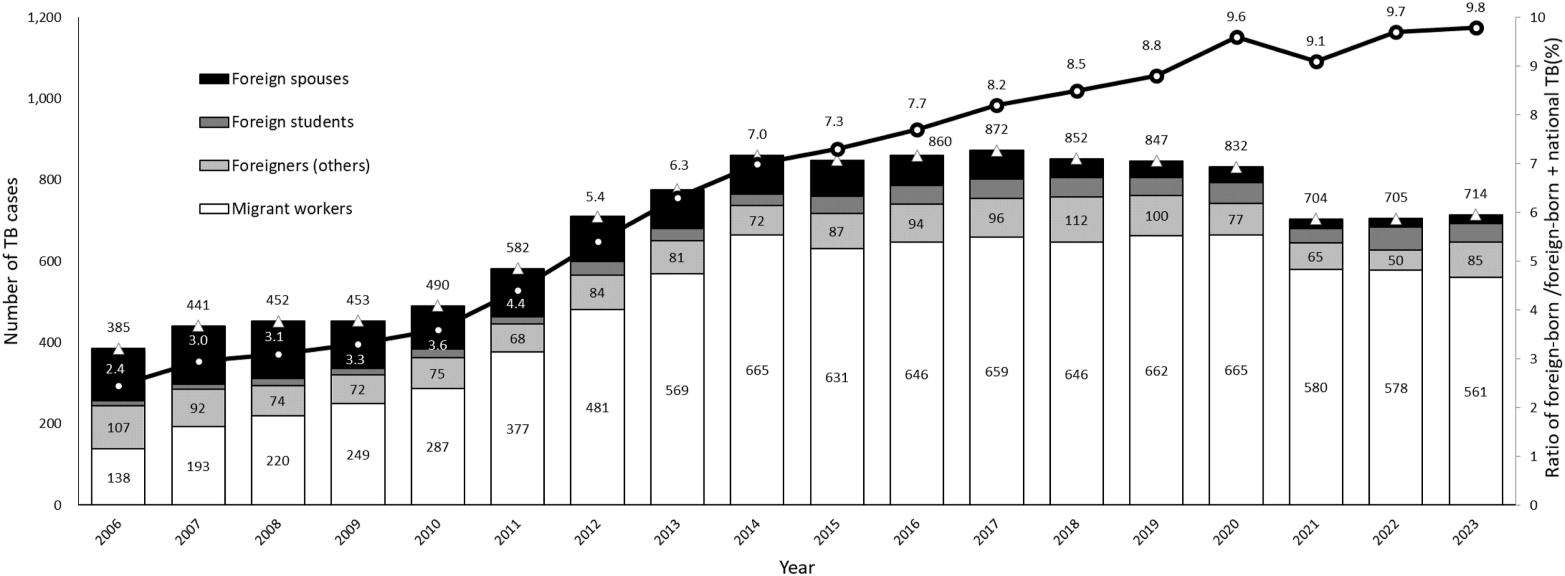
Foreign-born TB in 2006–2023 stratified by 4 types of residence permits, including foreign workers, foreign spouses, foreign students and foreigners (others) and the ratio of foreign-born TB compared to foreign-born and Taiwanese national TB. Clear triangles = total number of foreign-born TB; clear circles = the proportion of foreign-born TB of foreign-born and Taiwanese national TB (%).

**Figure 3.**
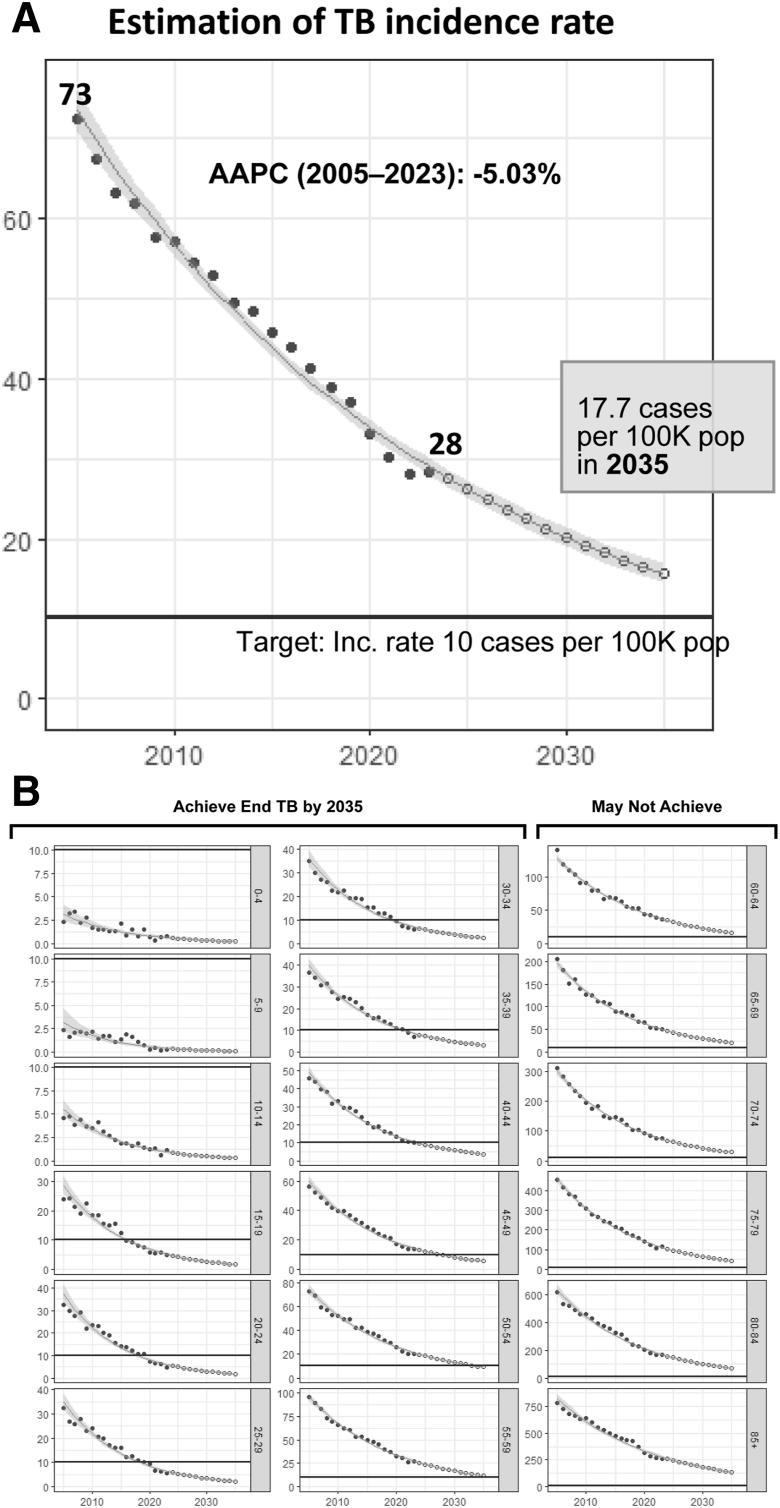
**A**: The projected TB incidence towards 2035 with the trend fitting by TB incidence during 2005–2023. To predict TB incidence from 2024 to 2035, an interrupted time series model was used. A negative binomial distribution was assumed for annual number of newly confirmed TB. Due to the rapidly aging society in Taiwan, the prediction for crude TB incidence for all age in 2035 is further adjusted to account for the age distribution expected in that year and the adjusted TB incidence for all ages is therefore 17.7/100,000 population. **B:** The projected TB incidence towards 2035 with age stratifications during 2005-2023. Black circles = observed data 2005–2023; clear circles = estimation 2024–2035; shaded area = trend and 95% CI; AAPC = average annual percent change; Inc = incidence; pop = population.

In 2023, for the first time, TB incidence remained unchanged for two consecutive years. This stabilization occurred as the number of medical visits and sputum examinations normalized to the pre-pandemic level,^23^ with the 2024 mid-year TB notifications resuming a 6% decline compared to 2023. The age-specific incidence trends (2005–2023) show the highest TB rates in individuals aged 85 and above, with a decline from 780.6 per 100,000 in 2005 to 261.0 in 2023 (Figure 1). Among those aged under 15, the TB incidence has been below 1 case per 100,000 since 2020, reaching 6 cases per million in 2023. The TB incidence among individuals under 40 years achieved the WHO End TB Strategy goal of <10/100,000 cases in 2023. Migrant workers are the largest contributors to foreign-born TB notifications in Taiwan besides foreign spouses, students and foreigners (others) (Figure 2). With the rising demands of labor, the total number of migrant workers exceeded 700,000 in 2023. Since 2020, the ratio of TB cases among foreign-born to those among foreign-born and citizens has exceeded 9%. Projections based on 2005–2023 data estimate Taiwan’s TB incidence will decline to 17.7/100,000 by 2035 adjusted by 2035 predicted population without innovative tools or strategies (Figure 3A).^8^ Although TB incidence among those below 60 is expected to meet End TB targets by 2035, it is unlikely for those aged over 60 (Figure 3B). TB elimination (<1 case per million population) appears achievable for young age groups (0–14 years) by 2035.

## INTERVENTIONS FROM 2005–2024 AND THEIR IMPACT

### Laboratory services, reporting and recording and collaboration with universal health care

Taiwan’s contracted and authorized laboratories, including all laboratories capable of performing MTBC culture and phenotypic drug-susceptibility testing (DST), have been quality-assured through routine proficiency testing since 2007, with on-demand onsite visits by Taiwan CDC.^24,25^ Since 2008, MDR-TB has been confirmed by the National Reference Laboratory (NRL), with genotypic DST routinely performed for rifampin resistant TB (RR-TB)/multi-drug resistant (MDR-TB) since 2017. Also, since 2014, Taiwan CDC required all TB laboratory testing data to be shared with the LARS. By 2018, automatic reporting and recording of TB cases were established through data exchange between LARS and the NIDRS. The auto-uploaded culture report coverage for new TB cases reached 98.2% in 2019 and 99.1% in 2023.

Using claimed data from the NHI (2005–2007), TB notification completeness and timeliness were 96.3%, with 80% of cases notified within 7 days of prescribing anti-TB medications, but only 77% of foreign TB patients were notified, of whom 92% were notified within 7 days.^26^ Previously notified TB cases also had a lower re-notification rate (86%) and timeliness (71%) during the same period.^26^ Performing three sets of sputum cultures routinely for presumptive TB patients increases bacteriological confirmation of TB or recurrent TB.^27^ Additionally, molecular drug resistance diagnostics are provided for high-risk patients, including those with prior TB treatment, TB preventive therapy (TPT), or known DR-TB exposure.

According to the Department of Labor, migrant workers in Taiwan are required to undergo health examinations within three days of arrival, at the 6^th^, 18^th^, and 30^th^ post-entry months, and annually thereafter. Prior to 2014, migrant workers diagnosed with TB were deemed unfit and repatriated.^28^ Regulatory changes in 2014 allowed workers to stay for treatment if employers consented to Directly Observed Therapy (DOT) beyond the post-arrival health examinations. In 2015, further amendments permitted migrant workers with TB upon arrival to stay as long as their employers agreed. These changes improved TB notification among foreigners from 89% in 2016 to 97% in 2020, with 96.5% of cases notified within 7 days.^29^ In 2022, further amendments allowed workers to stay for treatment based on their own consent, regardless of employer approval. As a result, the proportion of migrant workers staying for TB treatment increased from 10.6% in 2014 to 23.0% in 2015, 43.2% in 2016, and 88.8% in 2022.

### Patient support, contact tracing and examinations

Once a TB patient is diagnosed, treatment with support is essential to prevent further transmission and reduce mortality. In 2006, Taiwan CDC adopted a people-centered DOT program,^30^ assigning each patient a government-employed DOT worker, alongside a PHN and TB case manager, to provide treatment support. Feedback from DOT workers enabled PHNs and other entities to assist patients and reduced treatment interruption due to socio-economic challenges or adverse effects (AEs), significantly reducing loss to follow-up and TB-specific mortality.^30,31^ From 2008 to 2023, Taiwan maintained a recurrent rate below 1% (0.56–0.97%) for DS-TB patients. The DOT program, covering 98% of TB patients, relied primarily on in-person community-based DOT. In 2015, electronic DOT (eDOT), an app for video DOT, was introduced to address mobility, lifestyle and privacy concerns, though usage remained below 5% until the COVID-19 pandemic (2021–2022), when adoption rose to 33.5% for DS-TB and 28.5% for TBI contacts.^32^ Utilization reduced to 5-6% during mid-2023 but improved with the user-friendly eDOT version 2.0 launch in November 2023, reaching 16% usage by 2024.

To support low-income TB patients, the Taiwan Anti-Tuberculosis Association provides an annual USD 31,000 grant, aiding those without social welfare resources. In 2018, The 2018 National TB Patient Catastrophic Cost Survey revealed 22% of non-MDR-TB and 45% of MDR-TB households faced catastrophic costs due to TB (personal communication Lin HH), primarily due to non-medical expenses (49%), income loss (34%), and medical expenses (17%) before TB diagnosis. Contributing factors strongly associated with catastrophic costs, included low household income, TB stigma, and receiving inpatient treatment.

Following implementation of the DOTS program, efforts were intensified in contact investigation (CI), alongside the implementation of TPT. CI has long been part of TB control programs in Taiwan but registration of contacts only started after 2004.^33,34^ The average number of contacts per people living with TB was 2.2 in 2006. With DOT workers assisting PHNs in administering care and enabling PHNs to focus on CI, in 2007, the introduction of referral forms for CI to alleviate co-payment burdens for contact examinations, standardized tracing procedures, and training in interviewing skills further boosted CI and investigating TB clusters.^33^ By 2008, the average number of contacts per TB patients increased to 5.2, surpassing 10 by 2014 and reaching 14 in 2019. Despite COVID-19 disruptions, averages remained stable, with 12 in 2020–2021, 11 in 2022 and 12.5 in 2023.

### Program for DR-TB

**Figure 4.**
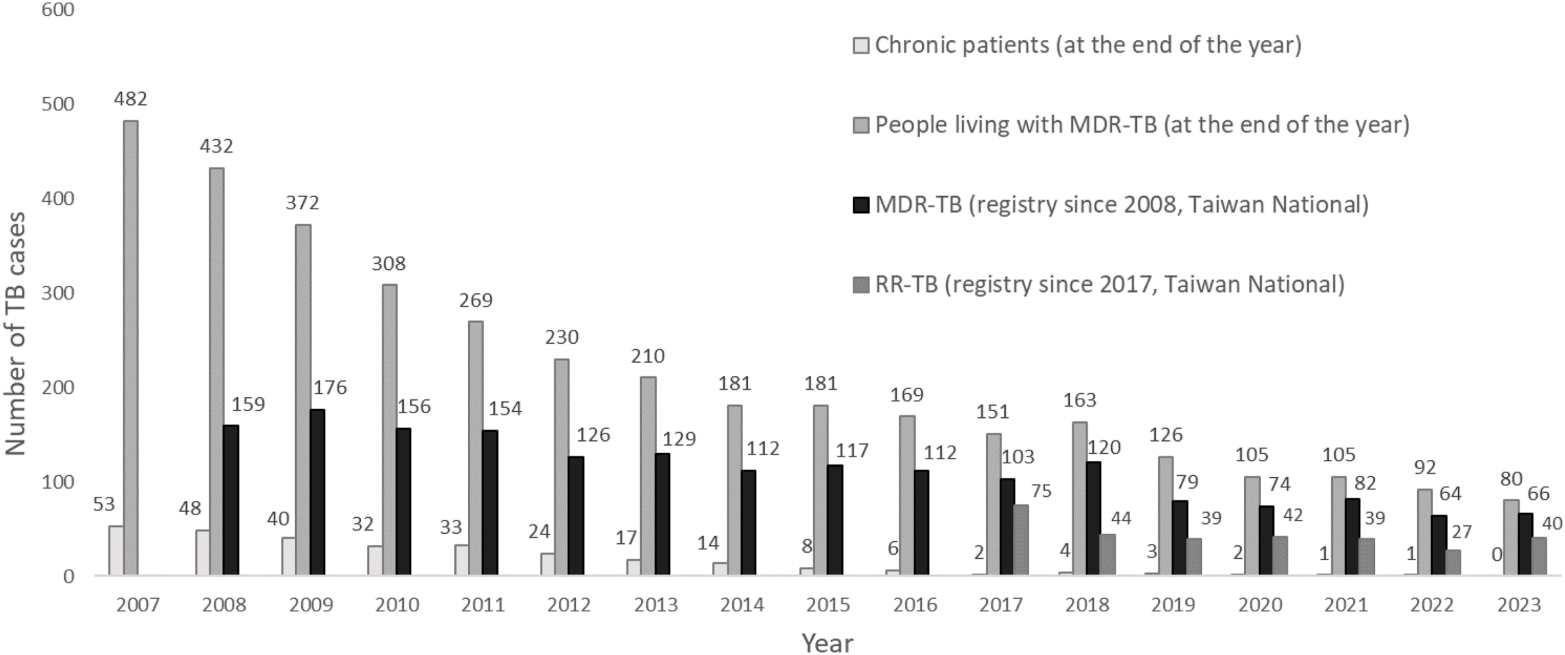
The trend number of multi-drug resistant TB (MDR-TB), rifampin resistant TB (RR-TB) without isoniazid resistance, chronic patients and people living with MDR-TB after implementation of patient-centered care, government-initiated hospital-based program for MDR-TB, the Taiwan MDR-TB Consortium, 2007–2023.

The Taiwan MDR-TB Consortium (TMTC), established in 2007, was created to enhance person-centered care for DR-TB, addressing the challenge of the 29% loss-to-follow-up rate in the 1990s.^35^ Approximately 25% of the NTP budget was allocated to TMTC, which supported individualized regimens for newly diagnosed MDR-TB cases and reevaluated ‘chronic-open’ patients, over 80% of whom lacked DST confirmation.^27,35^ In 2007, while the prevalence of MDR-TB cases was 482 (figure 4), sputum samples were recollected for DST, and patient records were reviewed, enabling comprehensive treatment. Despite the absence of newer drugs initially, the program ensured molecular diagnostics for high-risk groups, uninterrupted second-line drug supplies (fluoroquinolones and aminoglycosides), managing AEs, and incentives to support adherence over 18–24 months. These efforts reduced the loss-to-follow-up rate to 2.9% and recurrence rate to 0.6 per 1,000 person-years.^35-37^ In 2011, the program expanded to RR-TB and resistance to three first-line drugs other than rifampin, maintaining low recurrence rates of RR/MDR-TB at 0.34/0.35 per 1,000 person-years.^38^

Patients with fluoroquinolone resistance had poorer outcomes but better than those with both aminoglycoside and fluoroquinolone resistance (i.e., the old definition of XDR-TB).^35,36,38^ The introduction of bedaquiline and delamanid in 2015–2016 improved outcomes for XDR-TB and pre-XDR-TB,^39^ later expanding to MDR-TB/RR-TB patients in 2019 and to those intolerant to anti-TB treatment in 2022. In 2021, launch of the BPaLM/BPaL regimen shortened treatment to 6–9 months for XDR-TB and pre-XDR-TB and expanded to MDR-TB in 2023 and to RR-TB patients, as well as those intolerant to anti-TB treatment in 2024. By 2024, the BPaLM/BPaL regimen covered 89.7% of RR/MDR-TB patients receiving treatment. In 2017, additional advances included establishment of active TB drug safety monitoring and management (aDSM),^40^ and therapeutic drug monitoring (TDM) for key drugs since 2020. Despite these improvements, a 20% all-cause mortality rate during MDR-TB management highlights the need to address comorbidities in an aging population.

Since 2009, MDR-TB contacts received CXR screenings every 6 months for two years, with timely referrals for presumptive cases.^41^ A 9-month fluoroquinolone (9FQ) regimen, piloted in 2018 and expanded nationwide in 2021, faced 18.9% discontinuation due to AEs like joint pain. Greater access to TDM for levofloxacin adjustments and expanded care facilities is needed.^42^ Newly-diagnosed MDR-TB cases dropped from over 150 (2008–2011) to fewer than 130 by 2012 (within five years) and since 2017 the figure has dropped below 100. Prevalent MDR-TB cases declined from 482 in 2007 to 80 in 2023, while chronic patients decreased from 53 in 2007 to fewer than 5 in 2017 and zero in 2023 (Figure 4). This demonstrates that once an MDR-TB program is initiated, significant progress can be observed within a decade.

### Active case finding, TBI diagnosis and preventive treatment

**Figure 5.**
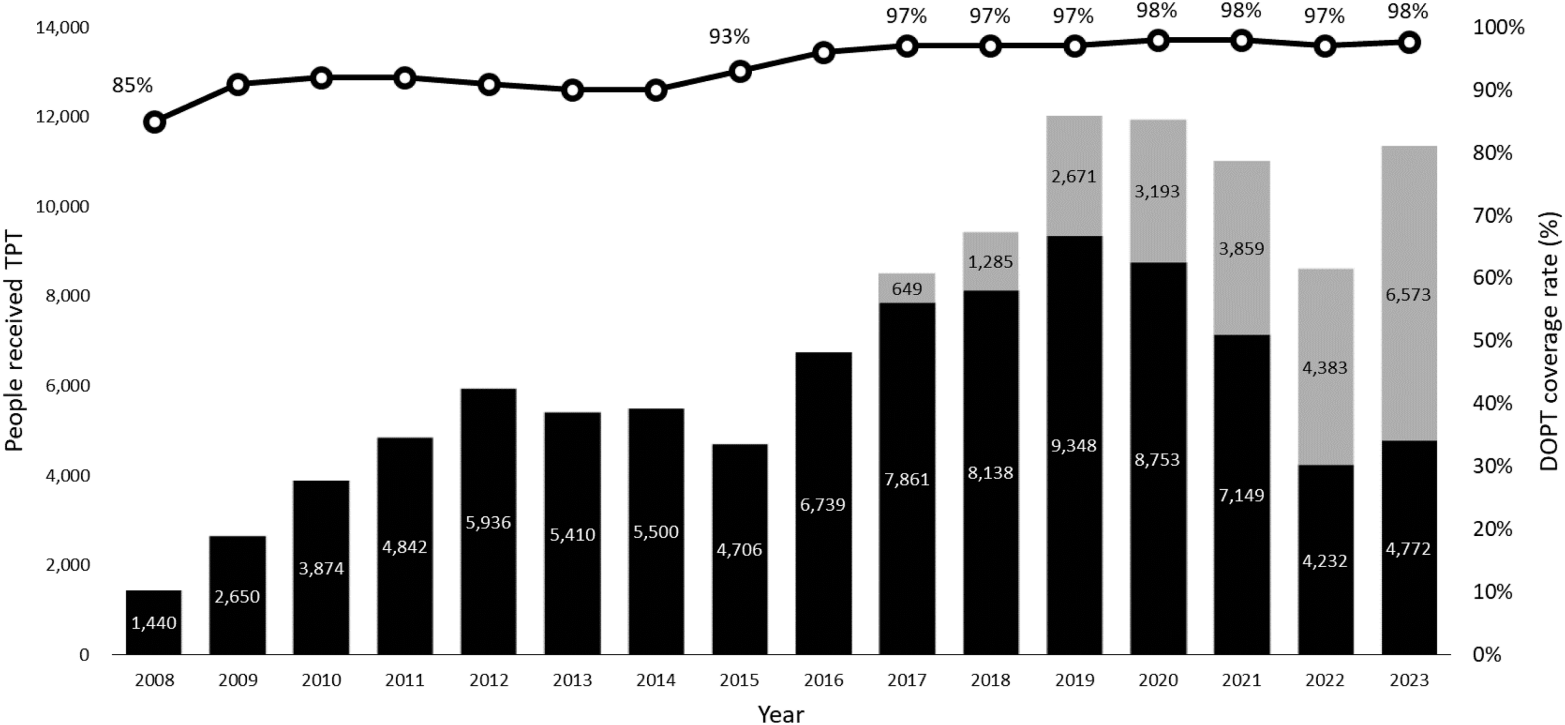
Uptake of TB infection treatment and coverage rate of directly observed preventive therapy. Black bar = No. of contacts with TBI received TPT; gray bar = No. of high-risk populations with TBI received TPT; clear circles = DOPT coverage rate (%) for TPT. TBI = TB infection; No. = number; TPT = TB preventive therapy; DOPT = directly observed preventive therapy.

The active case finding (ACF) program includes annual screening for healthcare workers (HCWs), employees and residents in LTCFs, and employees and inmates in correctional facilities. CXR screening is conducted for students entering high schools, colleges, and universities, as well as for newly onboarded employees and military personnel. Mobile CXR screening vans target vulnerable populations, including economically disadvantaged groups, elderly individuals in communities, and those with household registration in mountainous aboriginal areas (MARs). Overall, 4.0 % of new TB cases are detected through ACF programs annually. For eligible contacts (such as child contacts aged under 13, household contacts, or contacts with comorbidities for all culture-proven index TB patients, or contacts who shared 8 hours per day or a cumulative 40 hours during contagiousness period with smear-positive, culture-proven index patients), TBI testing is conducted using Tuberculin Skin Test (TST) for children aged under 2 and interferon-gamma release assay (IGRA) for those aged 2 and above.^43,44^ The overall number of people who received TPT and the proportion of DOPT they received are shown in Figure 5.

Since 2008, 9-month daily isoniazid (9H) was launched nationwide for contacts under 13 with positive TST.^45^ In 2016, IGRA was endorsed nationwide for contacts aged 5 and above alongside the 12-dose weekly isoniazid and rifapentine (3HP) regimen. Both rifapentine and 300 mg isoniazid were imported for public health utilization by Taiwan CDC and a pre-licensure post-marketing surveillance collaborating with Taiwan Food and Drug Administration and Taiwan Drug Relief Foundation was implemented to ensure the safety.^46^ By 2018, the TBI testing and treatment program expanded to LTCFs and MARs, complementary with pre-existing ACF program,^47–49^ and in 2020, to people living with HIV and inmates,^50,51^ alongside the endorsement of 4-month daily rifampin (4R) and 3-month daily isoniazid and rifampin (3HR).^52^ In 2021, IGRA testing was extended to contacts aged 2 and above, with a fixed-dose combination of 3HP introduced. The program further expanded to foreign spouses in 2022 and 1-month daily isoniazid and rifapentine (1HP) and 6-month daily isoniazid (6H) were adopted in 2023.^42^ In the second half of 2023, TBI testing and treatment in LTCFs became part of the NHI pay-for-performance (P4P) program, under the NHI Long-term Care Plan. This inter-departmental collaboration incentivized medical facilities to deliver outreached ACF, TBI diagnosis and treatment in LTCFs, and provided P4P to TPT among contacts and high-risk populations mentioned above, including further expansion to individuals with chronic obstructive lung disease (aged 60 years and above), dialysis patients, and poor controlled diabetics (HbA1c higher than 9% and aged 45 years and above).

## CRITICAL REFLECTIONS

The ITRP has made recommendations to align Taiwan’s NSP with evolving epidemiological trends, emerging technologies, and systemic challenges, reinforcing Taiwan’s commitment to global TB elimination.^13^ Nucleic acid amplification tests (NAATs) with higher sensitivity and shorter turnaround time than smear microscopy, differentiate MTBC and non-tuberculosis mycobacterium (NTM) and reduce treatment delays and pre-treatment death.^53,54^ A recent study revealed that the incremental yield of the third sputum smear in Taiwan was negligible.^55^ It may be reasonable to perform NAAT, smear and culture on the first specimen and culture alone on the second.^55^ WHO targets universal NAAT for all presumptive TB cases by 2027.^56^ Taiwan’s 2023 NAAT coverage was 74% (92% for smear-positive and 60% for smear-negative cases). Taiwan CDC will launch universal NAATs in 2025. Universal NAATs are also vital for timely DR-TB detection.^55^ Streamlined laboratory engagement has enhanced the information system's efficiency. Further automation of laboratory connection via a unified MoHW digital platform would accelerate reporting consistency and timeliness.

In 2023, individuals aged over 65 accounted for 85% of deaths during TB treatment, with <60% treatment success. To address the challenge of a super-aged society, Taiwan must enhance screening, prompt diagnosis, aDSM and integrate TB management with geriatric care.^1,2^ High TB mortality in the elderly stems from competing comorbidities and treatment discontinuation due to AEs.^1-3^ Preventing TB with TPT is crucial to reducing mortality and transmission in LTCFs.^57^ However, the elderly encountered 20% of permanent discontinuation due to AEs from TPT, leading to low treatment completion.^58,59^ For those aged 65 and above, in-person DOPT delivering aDSM and support, supplemented by eDOT 2.0 ensures person-centered care without overburdening services. Analyses distinguishing TB-related deaths from deaths caused by comorbidities are essential, along with better tracking of diagnostic delays focusing on patient delays. The gaps in social protection and household TB-related costs need to be monitored to break the ‘poverty-disease-poverty’ cycle.

Sustaining centers of excellence is essential for TB control but best practices for maintaining human resources in TB low-burden countries have rarely been reported,^60^ and it is challenging to attracting young physicians to focus on TB. Collaborating with the TMTC to create specialized centers for continued education, expert consultations, and addressing complex TB cases with comorbidities, AEs or drug-drug interactions, may help to maintain expertise. Regular refresher training for HCWs and exploring emerging technologies, such as clinical decision support systems powered by artificial intelligence (AI), may enhance TB care.

Pre-migration CXR screening of foreign workers need to be maintained, with TBI management strategies explored for this group.^4,61,62^ Deportation policies for foreign-born MDR-TB patients should be reevaluated, as they can deter early consultation and increase community transmission.^5^ Multilingual functionality in eDOT 2.0 could improve adherence among foreign-born TB patients. Addressing language barriers and strengthening education for migrants is critical for effective TB care.^5^ Legal policies must not discourage symptomatic individuals from seeking diagnosis. Comprehensive, patient-centered approaches for migrants and vulnerable groups can sustain progress toward TB elimination.^4,61,62^

Regarding ACF, high-risk groups for TB need to be clearly defined. Geo-spatial mapping and genotyping (such as whole genome sequence) can pinpoint high-burden areas for more targeted ACF initiatives. Portable digital CXR and AI-powered computer-aided detection tools are recommended for remote areas like MARs. Pre-employment IGRA screening for HCWs could establish baselines for future CI in medical settings. Achieving the End TB goals by 2035 requires intensified ACF and large-scale TBI.^63^ Current TBI programs remain limited, with a predictive model suggesting declines in TB incidence will primarily result from population aging without significant expansion.^64^ Manpower shortages and high turnover rates need to be addressed and AI-based tools may assist PHNs and TB case managers in handling complex cases.

Since 2016, Taiwan’s annual TB budget has remained steady at $32–33 million. However, it is possible that budgets will be cut, as happened in 1970s in the U.S.A., resulting in a resurgence of TB.^65^ Other challenges include the impacts of immigration, global epidemics like HIV/AIDS and COVID-19,^66^ and the severe public health disruptions that result from military conflict.^67,68^ Experiences from other countries demonstrate that TB elimination can be planned programmatically, with regional efforts playing a crucial role, supporting national and global elimination goals.^4,61,62,69,70^ To safeguard progress in TB control, Taiwan must maintain steady funding, invest in innovative tools, and implement strategies to retain expertise in TB care while also adapting to emerging challenges.

## CONCLUSION

The Taiwan NTP frequently draws on the experiences of other countries to avoid complacency regarding its moderate TB incidence rate. Taiwan has significantly improved early diagnosis and treatment by continually adopting new tools and medications, providing user-friendly platforms and implementing diverse policy interventions supported by operational research and incentives for targeted populations. Over the past two decades, free TB care has been provided universally, ACF and TBI treatment among contacts and high-risk groups have been expanded, and DR-TB cases have been properly managed. This approach has helped reduce TB incidence and mortality.

For future challenges we recommend utilizing comprehensive approaches (including AI tools) to deal with increasingly complex TB cases (due to aging and co-morbidities), to address TB/TBI among foreign workers, to mitigate the impact of an M-shaped society, to identify hot spots of ongoing transmission and drug resistance by universal genotyping, and to enhance information exchange and utilization through inter-departmental collaborations. Taiwan’s consistent investment in TB control and prevention reflects the strong political commitment to addressing this long-standing public health issue. This commitment will likely be the most crucial factor in achieving the goal of ending TB in the coming decade. With the support of resilient healthcare and public health systems, led by the Taiwan MoHW, we are well-positioned to combat TB effectively.

## References

[1] Caraux-Paz P, Tuberculosis in the Elderly. J Clin Med 2021;10(24):5888. 34945187 10.3390/jcm10245888 PMC8703289

[2] Pratt RH, Tuberculosis in older adults in the United States, 1993-2008. Journal of the American Geriatrics Society 2011;59(5):851-857. 21517786 10.1111/j.1532-5415.2011.03369.x

[3] Lee CS, The incidence of tuberculosis recurrence: Impacts of treatment duration of and adherence to standard anti-tuberculous therapy. J Infect Public Health 2023;16(11):1778-1783. 37738694 10.1016/j.jiph.2023.09.005

[4] Van den Boom M, Commitment, partnerships and operational research: three priorities for 11 EMR countries to achieve TB elimination. IJTLD Open 2024;1(1):50-55. 38919409 10.5588/ijtldopen.23.0470 PMC11189605

[5] World Health Organization. Innovative solutions for the elimination of tuberculosis among migrants and refugees. Report of the 7th edition of the World Innovation Summit for Health. Geneva, Switzerland: WHO, 2024.

[6] Department of Household Registration, Ministry of Interior, Taiwan. Household registration statistics data analysis in Dec 2023.

[7] World Population Review. Total fertility rate 2024.

[8] National Development Council, Taiwan. Population Projections for the R.O.C. (Taiwan).

[9] Centers for Disease Control, Ministry of Health and Welfare, Taiwan. Taiwan Tuberculosis Control Report. 2023.

[10] Ministry of Health and Welfare, Taiwan. National Mobilization Plan to Halve TB in 10 Years - Phase 2.

[11] Ministry of Health and Welfare, Taiwan. Taiwan's End Tuberculosis (TB) by 2035 Project Phase II.

[12] Centers for Disease Control, Ministry of Health and Welfare, Taiwan. External review of “Halving TB in 10 years program in Taiwan, 2006 – 2015”.

[13] Centers for Disease Control, Ministry of Health and Welfare, Taiwan. External Review of “Taiwan’s End TB by 2035 Project Phase II”.

[14] Ministry of Health and Welfare, Taiwan. Article 39 and 64 in the Communicable Disease Control Act.

[15] Chiang CY, The impact of national health insurance on the notification of tuberculosis in Taiwan. Int J Tuberc Lung Dis 2002;6(11):974-979. 12475143

[16] Lo HY, Trends in tuberculosis in Taiwan, 2002-2008. J Formos Med Assoc 2011;110(8):501-510 21783019 10.1016/S0929-6646(11)60076-4 PMC7134926

[17] Chen CC, Health system delay among patients with tuberculosis in Taiwan: 2003-2010. BMC Infect Dis 2015;15:491. 26527404 10.1186/s12879-015-1228-x PMC4629405

[18] National Health Insurance Administration, Ministry of Health and Welfare, Taiwan. National Health Insurance Open Data Sharing Platform. National Health Insurance Statistics Section. Trends in National Health Insurance Statistics.

[19] Li YH, The effects of pay-for-performance on tuberculosis treatment in Taiwan. Health Policy Plan 2010;25(4):334-341. 20207703 10.1093/heapol/czq006

[20] Lee CY, Using financial incentives to improve the care of tuberculosis patients. Am J Manag Care 2015;21(1):e35-42. 25880266

[21] Chen TC, Fluoroquinolones are associated with delayed treatment and resistance in tuberculosis: a systematic review and meta-analysis. Int J Infect Dis 2011;15(3):e211-6. 21195001 10.1016/j.ijid.2010.11.008

[22] Shen GH, Does empirical treatment of community-acquired pneumonia with fluoroquinolones delay tuberculosis treatment and result in fluoroquinolone resistance in Mycobacterium tuberculosis? Controversies and solutions. Int J Antimicrob Agents 2012;39(3):201-5. 22285045 10.1016/j.ijantimicag.2011.11.014 PMC7127649

[23] Chu PW, Impact of the COVID-19 Epidemic on TB Diagnosis and Transmission in Taiwan. E poster at the 52nd Union World Virtual Conference on Lung Health, 2021.

[24] Wu MH, External quality assessment of sputum smear microscopy in Taiwan. Int J Tuberc Lung Dis 2009;13(5):606-12. 19383194

[25] Wu MH, Proficiency of drug susceptibility testing for Mycobacterium tuberculosis in Taiwan, 2007-2011. Int J Tuberc Lung Dis 2013;17(1):113-9. 23232011 10.5588/ijtld.12.0521

[26] Lo HY, Completeness and timeliness of tuberculosis notification in Taiwan. BMC Public Health 2011;11:915. 22151346 10.1186/1471-2458-11-915 PMC3260335

[27] Lee PH, Impact of universal drug susceptibility testing and effective management of multidrug-resistant tuberculosis in Taiwan. PLoS One 2019;14(4):e0214792. 30939150 10.1371/journal.pone.0214792 PMC6445419

[28] Bai KJ, Tuberculosis among foreign-born persons in Taiwan, 2002-2005. J Formos Med Assoc 2008;107(5):389-395. 18492623 10.1016/S0929-6646(08)60104-7

[29] Lo HY, TB notifications among citizens and non-citizens in Taiwan. Int J Tuberc Lung Dis 2024;28(7):328-334. 38961552 10.5588/ijtld.23.0567

[30] Bloss E, Increasing directly observed therapy related to improved tuberculosis treatment outcomes in Taiwan. Int J Tuberc Lung Dis 2012;16(4):462-467. 22640512 10.5588/ijtld.11.0121

[31] Yen YF, Directly observed therapy reduces tuberculosis-specific mortality: a population-based follow-up study in Taipei, Taiwan. PLoS One 2013;8(11):e79644. 24278152 10.1371/journal.pone.0079644 PMC3838349

[32] Chen JJ, The Evolution of Electronic Directly Observed Therapy (eDOT) in Tuberculosis Treatment: Before, During, and After the COVID-19 Pandemic in Taiwan. Oral abstract presented at the 54^th^ Union World Conference of Lung Health in 2023, Paris, France.

[33] Chan PC, Huang LM, Suo J. It is time to deal with latent tuberculosis infection in Taiwan. J Formos Med Assoc 2009;108(12):901-3. 20040453 10.1016/s0929-6646(10)60001-0

[34] Ling DL, Contact investigation for tuberculosis in Taiwan contacts aged under 20 years in 2005. Int J Tuberc Lung Dis 2011;15(1):50-5. 21276296

[35] Chan PC, Taiwan Multidrug-Resistant Tuberculosis Consortium-TMTC. Effectiveness of a government-organized and hospital-initiated treatment for multidrug-resistant tuberculosis patients–a retrospective cohort study. PLoS One 2013;8(2):e57719. 23451263 10.1371/journal.pone.0057719 PMC3581541

[36] Yu MC, Treatment Outcomes of Multidrug-Resistant Tuberculosis in Taiwan: Tackling Loss to Follow-up. Clin Infect Dis 2018;67(2):202-210. 29394358 10.1093/cid/ciy066 PMC6030934

[37] Chen MY, Recurrence after Successful Treatment of Multidrug-Resistant Tuberculosis in Taiwan. PLoS One 2017;12(1):e0170980. 28125692 10.1371/journal.pone.0170980 PMC5270331

[38] Lee PH, Epidemic trend and prognosis analysis of drug-resistant tuberculosis. MOHW110-CDC-C-315-122110 Scientific research granted by Taiwan MoHW in 2021.

[39] Huang YW, Mitigating treatment failure of pulmonary pre-extensively drug-resistant tuberculosis: The role of new and repurposed drugs. J Microbiol Immunol Infect 2024;57(4):617-628. 38705821 10.1016/j.jmii.2024.04.008

[40] Lin CJ, Clofazimine and QT prolongation in the treatment of rifampicin-resistant tuberculosis: Findings of aDSM in Taiwan. J Microbiol Immunol Infect 2024;57(5):791-800. 39160114 10.1016/j.jmii.2024.08.002

[41] Lee PH, Risk of developing active MDR-TB diseases among contacts in Taiwan, 2016-2018. e-Poster at the 51^st^ Union World Virtual Conference on Lung Health, 2020.

[42] Chan PC, Assessment of Safety and Effectiveness of Ultra-Short 1HP and 9FQ for Latent Tuberculosis Infection Treatment. MOHW113-CDC-C-315-112103 Scientific research granted by Taiwan MoHW in 2024.

[43] Chan PC, Yang CH, Chang FY. Scaling up of latent tuberculosis infection treatment for close contacts of tuberculosis in Taiwan. J Formos Med Assoc 2011;110(12):733-736. 22351971 10.1016/j.jfma.2011.11.001

[44] Chan PC, Risk for tuberculosis in child contacts: development and validation of a predictive score. Am J Respir Crit Care Med 2014;189(2):203-213 24304428 10.1164/rccm.201305-0863OC

[45] Chan PC, Long-term Follow-up for Prevention of Tuberculosis among Contacts Receiving 6-month-isoniazid and 9-month-isoniazid—an Observation Cohort Study for Effectiveness. Oral abstract at the 53^rd^ Union World Virtual Conference on Lung Health, 2022.

[46] Chan PC, Pharmacovigilance for Public Health Utility: Safety Monitoring of New Regimen for Latent Tuberculosis Infection Treatment. Respirology 2018;23(S2):pp.5

[47] Chiu TF, Determinants of latent tuberculosis infection and treatment interruption in long-term care facilities: A retrospective cohort study in Taiwan. J Microbiol Immunol Infect 2022;55(6 Pt 2):1310-1317. 34686442 10.1016/j.jmii.2021.09.013

[48] Lee PH, Effectiveness of latent tuberculosis infection treatment among residents in long-term care facilities, Taiwan. Oral presentation at 54^th^ The Union World Conference on Lung Health, Paris, France, 2023.

[49] Liao YY, A public-private mix program to improve detection of tuberculosis in mountainous aboriginal regions in Taiwan. E poster at 53^rd^ The Union World Virtual Conference on Lung Health, 2022.

[50] Lin KY, Treatment responses to Integrase strand-transfer inhibitor-containing antiretroviral regimens in combination with short-course rifapentine-based regimens for latent tuberculosis infection among people with HIV. Clin Infect Dis 2024;78(5):1295-1303. 38051646 10.1093/cid/ciad730

[51] Lee PH, Prevalence of Latent Tuberculosis Infection and the Associated Factors in Correctional Institutions and Prisons in Taiwan, 2019 – 2023. Short oral presentation at the 54^th^ Union World Conference of Lung Health, Bali, Indonesia, 2024.

[52] Chan PC, Uptake of 4-month Rifampin Regimen among Isoniazid Resistant Index’s Contacts and Their Outcome of Treatment. E poster at the 52^nd^ Union World Virtual Conference on Lung Health, 2021.

[53] Huang WC, Performance of Nucleic Acid Amplification Tests in Patients with Presumptive Pulmonary Tuberculosis in Taiwan. Infect Dis Ther 2022;11(2):871-885. 35254635 10.1007/s40121-022-00610-2 PMC8900096

[54] Feng JY, Nucleic acid amplification tests reduce delayed diagnosis and misdiagnosis of pulmonary tuberculosis. Sci Rep 2022;12(1):12064. 35835940 10.1038/s41598-022-16319-8 PMC9283405

[55] Chiang CY, Incremental yield of serial sputum examinations in the diagnosis of pulmonary tuberculosis in Taiwan: Findings of a pragmatic trial. J Microbiol Immunol Infect 2023;56(6):1245-1252. 37802687 10.1016/j.jmii.2023.09.006

[56] World Health Organization. Global targets set in 2023 at the second UN high-level meeting on TB. In: The second United Nations high-level meeting on TB: new global pledge to end the TB epidemic.

[57] Getahun H, Management of latent Mycobacterium tuberculosis infection: WHO guidelines for low tuberculosis burden countries. Eur Respir J 2015;46(6):1563-1576 26405286 10.1183/13993003.01245-2015 PMC4664608

[58] Chan PC, Tolerability of rifapentine-based regimens in latent tuberculosis infection treatment in the elderly. Eur Respir J 2019 ;53(3):1802396. 30886023 10.1183/13993003.02396-2018

[59] Huang HL, Completion rate and safety of programmatic screening and treatment for latent tuberculosis infection in elderly patients with poorly controlled diabetic mellitus: a prospective multicenter study. Clin Infect Dis 2021;73(6):e1252-e1260. 33677558 10.1093/cid/ciab209 PMC8442788

[60] Wu S, Roychowdhury I, Khan M. Evaluations of training programs to improve human resource capacity for HIV, malaria, and TB control: a systematic scoping review of methods applied and outcomes assessed. Trop Med Health 2017;45:16. 28680324 10.1186/s41182-017-0056-7 PMC5493875

[61] Zureigat H, Steps towards TB elimination in Jordan. Int J Tuberc Lung Dis 2023;27(7):576-577. 37353864 10.5588/ijtld.23.0125

[62] Yaacoub H, Planning for TB elimination in Lebanon. Int J Tuberc Lung Dis 2023;27(3):171-174. 36855041 10.5588/ijtld.22.0673

[63] World Health Organization. Latent Tuberculosis Infection: Updated and Consolidated Guidelines for Programmatic Management. Geneva, Switzerland: WHO; 2018. 30277688

[64] Murray CJ, Salomon JA, Ortblad K. Global tuberculosis incidence and mortality during 1990–2013. Lancet 2015;385(9974):117-121.25530442

[65] Taylor Z, Nolan CM, Blumberg HM; American Thoracic Society; Centers for Disease Control and Prevention; Infectious Diseases Society of America. Controlling tuberculosis in the United States. Recommendations from the American Thoracic Society, CDC, and the Infectious Diseases Society of America. MMWR Recomm Rep 2005;54(RR-12):1-81. 16267499

[66] McQuaid CF, The impact of COVID-19 on TB: a review of the data. Int J Tuberc Lung Dis 2021;25(6):436-446. 34049605 10.5588/ijtld.21.0148 PMC8171247

[67] Topluoglu S, Taylan-Ozkan A, Alp E. Impact of wars and natural disasters on emerging and re-emerging infectious diseases. Front Public Health 2023;11:1215929. 37727613 10.3389/fpubh.2023.1215929 PMC10505936

[68] Akihiro Seita. “Think PHC, Do TB” Integration-based scale up of tuberculosis control in Japan.

[69] Jankovic Makek M, New priorities for Croatia to pursue tuberculosis pre-elimination. Eur Respir J 2024;63(6):2400279. 38901891 10.1183/13993003.00279-2024

[70] Commiesie E, Challenges and opportunities for TB elimination in Suriname. Int J Tuberc Lung Dis 2023;27(12):946-948. 38042972 10.5588/ijtld.23.0309

